# Association between nocturnal blood pressure dipping patterns and left atrial reservoir strain in untreated adults with office-defined grade 1 essential hypertension

**DOI:** 10.3389/fcvm.2026.1898739

**Published:** 2026-07-27

**Authors:** Lei Zha, Haoqin Zhu, Jiajia Liu, Lianghui Wan, Yanzhe Ma, Wei Liu

**Affiliations:** 1 Department of Cardiology, Tianjin Chest Hospital, Tianjin, China; 2 Department of Cardiology, Chengdu Second People’s Hospital, Chengdu, China; 3 Department of Cardiology, Second Affiliated Hospital of Tianjin University of Traditional Chinese Medicine, Tianjin, China

**Keywords:** ambulatory blood pressure monitoring, hypertension, left atrial reservoir strain, nocturnal dipping, speckle-tracking echocardiography

## Abstract

**Background:**

Abnormal nocturnal blood pressure (BP) profiles may expose the left atrium to persistent hemodynamic load before overt remodeling occurs. This study evaluated the association between nocturnal systolic blood pressure (SBP) dipping patterns and left atrial reservoir strain (LARS) in untreated adults with office-defined grade 1 essential hypertension.

**Methods:**

This single-center retrospective cross-sectional study enrolled 315 adults who underwent 24-h ambulatory blood pressure monitoring (ABPM) and transthoracic echocardiography within 7 days. BP dipping patterns were classified as dipper, non-dipper, reverse-dipper, or extreme-dipper. Continuous LARS was the primary outcome; low LARS (<35%) was the operational secondary outcome. The primary model was adjusted for age, sex, body mass index (BMI), 24-h SBP, fasting plasma glucose, natural-log-transformed triglycerides, low-density lipoprotein cholesterol (LDL-C), high-density lipoprotein cholesterol (HDL-C), and estimated glomerular filtration rate (eGFR).

**Results:**

There were 126 dippers (40.0%), 124 non-dippers (39.4%), 45 reverse-dippers (14.3%), and 20 extreme-dippers (6.3%); 300 participants (95.2%) met ambulatory criteria for sustained hypertension, and 100 (31.7%) had low LARS. LARS differed across patterns (*P* < 0.001). Compared with dippers, non-dippers and reverse-dippers had lower adjusted LARS [*β* = −3.44, 95% confidence interval (CI) −4.68 to −2.20; *β* = −5.92, 95% CI −7.80 to −4.04] and higher odds of low LARS [non-dippers odds ratio (OR) = 3.68, 95% CI 1.83–7.39; reverse-dippers OR = 8.12, 95% CI 3.00–21.98; both *P* < 0.001]. Extreme-dipping was not associated with either outcome. When nighttime SBP replaced 24-h SBP, the associations were attenuated but remained statistically significant; the direction of association did not change in analyses restricted to participants with complete sleep diaries or sustained hypertension. Restricted cubic spline (RCS) analysis suggested nonlinearity.

**Conclusion:**

Non-dipping and reverse-dipping BP patterns were associated with lower LARS and a higher likelihood of low LARS. Prospective multicenter studies are needed to determine prognostic and incremental clinical value.

## 1 Introduction

Elevated blood pressure (BP) is an important modifiable risk factor for cardiovascular disease and mortality burden in adults (1). This study focused on adults whose office BP was within the grade 1 hypertension range, who had not received antihypertensive therapy, and who had no established clinical cardiovascular disease. Nocturnal BP regulation is influenced by sleep-wake rhythm, the autonomic nervous system, and humoral factors (2). Elevated nighttime BP, insufficient dipping, or reverse dipping is associated with hypertension-mediated organ damage and cardiovascular outcomes (3). Twenty-four-hour ambulatory blood pressure monitoring (ABPM) can characterize 24-h, awake, and asleep BP and identify white-coat, masked, and abnormal dipping phenotypes (4).

Relative BP dipping patterns and absolute nighttime BP load do not convey identical information and should be distinguished in analyses and interpretation (5). Left atrial reservoir strain (LARS) quantitatively evaluates left atrial expansion and reservoir function during left ventricular systole and is sensitive to diastolic load and early atrial remodeling (6). Recent evidence in hypertensive populations suggests that left atrial strain is lower in non-dippers than in dippers, indicating that circadian rhythm abnormalities may be associated with subclinical left atrial functional changes (7).

Population data from two-dimensional speckle-tracking echocardiography (2D-STE) provide LARS reference ranges stratified by age and sex (8). Left atrial volume index (LAVI), the ratio of mitral early diastolic E-wave velocity to mitral annular early diastolic e′ velocity (E/e′), and left atrial strain assess left atrial function from complementary dimensions of structure, filling pressure, and atrial mechanics (9). Existing evidence is heterogeneous in treatment status, BP grade, equipment, and analysis software, which limits direct extrapolation to an untreated office-defined grade 1 hypertension population (10). This study aimed to evaluate the association between nocturnal BP dipping patterns and LARS in this population.

## 2 Materials and methods

### 2.1 Study design and participants

This was a single-center retrospective cross-sectional study. Records of patients who completed 24-h ABPM and transthoracic echocardiography between 1 January 2021 and 31 December 2025 were consecutively retrieved. Office-defined grade 1 essential hypertension was determined by standardized office BP measurements on at least two separate dates, with systolic blood pressure (SBP) of 140–159 mmHg and/or diastolic blood pressure (DBP) of 90–99 mmHg (11). Untreated status was defined as no previous antihypertensive therapy and no use of medications that could substantially affect BP levels before the examinations. Inclusion criteria were age 18–75 years, valid ABPM, an ABPM-to-echocardiography interval of ≤7 days, sinus rhythm, and available apical four-chamber and two-chamber views suitable for 2D-STE analysis. Exclusion criteria included secondary or suspected secondary hypertension, established cardiovascular disease, moderate-to-severe valvular disease, significant arrhythmia or pacemaker implantation, left ventricular ejection fraction (LVEF) <50%, diabetes mellitus, estimated glomerular filtration rate (eGFR) <60 mL/min/1.73 m^2^ or other major systemic disease, and moderate-to-severe obstructive sleep apnea (OSA) previously diagnosed by sleep-specialist evaluation or polysomnography. Systematic OSA screening was not performed for all patients during the study period; body mass index (BMI) data were available for all 315 participants, whereas the Epworth Sleepiness Scale and nocturnal oxygen saturation were not systematically recorded.

**Figure 1:**
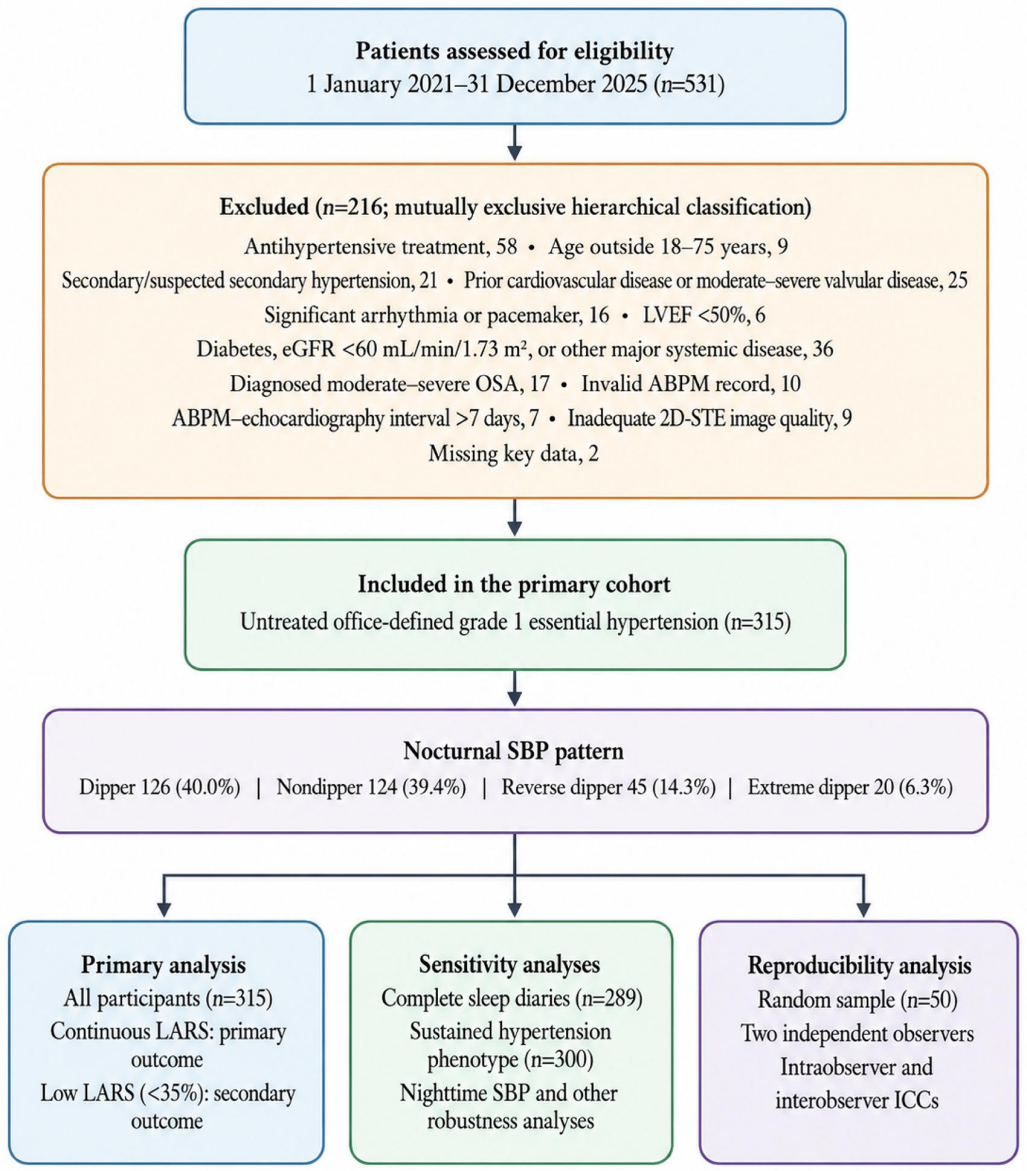
Participant selection flow. During the study period, 531 patients were screened. According to prespecified hierarchical and mutually exclusive rules, 216 were excluded, and 315 untreated patients with office-defined grade 1 essential hypertension entered the main analysis. The complete-sleep-diary, sustained-hypertension phenotype, and reproducibility subsets included 289, 300, and 50 patients, respectively. ABPM, 24-h ambulatory blood pressure monitoring; eGFR, estimated glomerular filtration rate; ICC, intraclass correlation coefficient; LARS, left atrial reservoir strain; LVEF, left ventricular ejection fraction; OSA, obstructive sleep apnea; SBP, systolic blood pressure; *n*, number of patients; 2D-STE, two-dimensional speckle-tracking echocardiography.

During the study period, 531 patients were screened. According to prespecified hierarchical and mutually exclusive rules, 216 were excluded: previous or current antihypertensive therapy in 58, age outside the 18–75-year range in 9, secondary or suspected secondary hypertension in 21, previous cardiovascular disease or moderate-to-severe valvular disease in 25, significant arrhythmia or pacemaker implantation in 16, LVEF <50% in 6, diabetes mellitus, eGFR <60 mL/min/1.73 m^2^, or other systemic disease in 36, diagnosed moderate-to-severe OSA in 17, invalid ABPM recording in 10, ABPM-to-echocardiography interval >7 days in 7, insufficient 2D-STE image quality in 9, and missing key data in 2. The final cohort included 315 untreated patients with office-defined grade 1 essential hypertension (Figure 1). The study was approved by the institutional ethics committee; the requirement for written informed consent was waived because deidentified historical data were used.

### 2.2 Clinical data and office blood pressure

Age, sex, height, weight, BMI, smoking, alcohol consumption, heart rate, and laboratory measurements were collected from electronic medical records. Office BP was measured according to standardized procedures for preparation, posture, cuff selection, and repeated measurements (12). An Omron HBP-1320 electronic BP monitor (Omron Healthcare, Kyoto, Japan) was used; this device has been validated according to the American National Standards Institute/Association for the Advancement of Medical Instrumentation/International Organization for Standardization (ANSI/AAMI/ISO) 81060-2:2013 and the 2010 revision of the European Society of Hypertension International Protocol (ESH-IP 2010) (13). BP was measured in both arms at the first assessment, and subsequent measurements were performed on the arm with the higher SBP. Within 30 min before measurement, patients avoided smoking, caffeine intake, and vigorous exercise and emptied the bladder. Patients rested in a seated position for ≥5 min with back and feet supported, legs uncrossed, and the upper arm supported at heart level. Mid-upper-arm circumference was measured, and the cuff was selected according to the device instructions; when arm circumference was at the boundary between two sizes, the larger cuff was used. Three consecutive measurements were taken at 1-min intervals, and the mean of the last two readings was used. The device model remained unchanged during the study period and was calibrated at least annually according to prespecified quality-control procedures.

### 2.3 Twenty-four-hour ambulatory blood pressure monitoring and dipping patterns

All patients underwent ABPM using a Microlife WatchBP O3 device (device log model: BP3MZ1-1; Microlife AG, Widnau, Switzerland); the accuracy validation of this platform is cited in the referenced study (14). ABPM implementation and quality control followed adult ABPM guidance (15). Before ABPM, mid-upper-arm circumference was measured, and the cuff was selected according to the manufacturer's applicable range. The cuff was usually placed on the non-dominant arm; when the inter-arm SBP difference was ≥10 mmHg, it was placed on the arm with the higher BP. After device fitting, a test measurement was performed to confirm cuff fit, device function, and patient tolerance. Measurements were taken every 20 min from 06:00 to 21:59 and every 30 min from 22:00 to 05:59. The main analysis used sleep diaries to define awake and asleep periods; fixed time windows of 06:00–22:00 and 22:00–06:00 were used for 26 patients with incomplete sleep diaries. A valid recording required duration ≥24 h, an overall valid reading proportion ≥70%, at least 20 valid awake readings, at least 7 valid asleep readings, and no interval >2 h without a valid reading. Before grouping, readings with device error codes, obvious cuff displacement or strenuous activity, and physiologically implausible values (SBP <70 or >250 mmHg, DBP <40 or >150 mmHg, or pulse pressure <20 or >150 mmHg) were excluded; no imputation was performed. The nighttime SBP dipping rate was calculated as (daytime mean SBP − nighttime mean SBP)/daytime mean SBP × 100%. Dipping patterns were classified as dipper (≥10% and <20%), non-dipper (≥0% and <10%), reverse-dipper (<0%), and extreme-dipper (≥20%). The sustained-hypertension phenotype was defined by meeting any of the following ambulatory BP thresholds: 24-h mean BP ≥130/80 mmHg, awake mean BP ≥135/85 mmHg, or asleep mean BP ≥120/70 mmHg. Patients with office grade 1 hypertension who did not meet any of these thresholds were classified as having a white-coat hypertension phenotype. The ABPM model, analysis software, and export rules remained consistent during the study period, and the device was calibrated at least annually.

### 2.4 Echocardiography and left atrial strain

Conventional cardiac chamber measurements were performed according to recommendations for adult cardiac chamber quantification (16), and diastolic function variables were obtained according to current echocardiographic recommendations (17). Transthoracic echocardiography was performed using a GE Vivid E95 system and an M5Sc-D transducer (GE HealthCare, Horten, Norway). Patients rested in the left lateral decubitus position for ≥10 min, and electrocardiography was recorded during the examination. Dedicated left atrial apical four-chamber and two-chamber views were acquired at end-expiration while avoiding left atrial foreshortening, with a frame rate of 60–80 frames/s over ≥3 consecutive sinus beats. All Digital Imaging and Communications in Medicine (DICOM) cine loops were analyzed offline using the same EchoPAC PC v204.0 software. The R wave was used as the zero reference point. The left atrial endocardial border was manually traced, and the automatically generated region of interest was adjusted while excluding the pulmonary vein ostia and left atrial appendage. The average of 3 consecutive cardiac cycles was obtained for each view, and the biplane mean was used as the patient-level value. Patients with unreliable tracking in either view were excluded from the analysis. Acquisition and analysis followed current strain consensus recommendations (18).

Lianghui Wan (Department of Cardiology, Tianjin Chest Hospital; 6 years of experience in echocardiography and 2D-STE analysis) completed all primary analyses; Yanzhe Ma (Department of Cardiology, Tianjin Chest Hospital; 5 years of experience in echocardiography and 2D-STE analysis) completed independent reproducibility measurements. Both observers were trained using the same analysis protocol and were blinded to clinical data, ABPM results, and BP dipping patterns. A computer-generated random sequence was used to select 50 patients. Four weeks after the initial analysis, Lianghui Wan repeated measurements using deidentified, re-randomized image sequences in which previous contours were not visible. Yanzhe Ma independently measured the same images and had no access to the primary observer's results. Intraobserver agreement was assessed using a two-way mixed-effects, absolute-agreement, single-measure intraclass correlation coefficient (ICC) [ICC(A,1)]; interobserver agreement was assessed using a two-way random-effects, absolute-agreement, single-measure ICC [ICC(2,1)].

Continuous LARS was the primary outcome. On the basis of 2D-STE evidence and the use of a 35% cutoff in a recent diastolic function assessment framework (19), LARS <35% was prespecified as the operational secondary outcome. A sensitivity analysis using the alternative threshold of LARS <30% was also performed. Interpretation of the 35% cutoff was limited to the acquisition platform, software version, R-wave reference point, and analysis workflow used in this study.

### 2.5 Renal function calculation

eGFR was calculated using the Chronic Kidney Disease Epidemiology Collaboration (CKD-EPI) 2021 creatinine equation without a race variable (20).

### 2.6 Statistical analysis

Analyses were performed using IBM SPSS Statistics 26.0 and R 4.3.0. The distributions of continuous variables were evaluated using the Shapiro–Wilk test together with quantile-quantile (Q-Q) plots. Approximately normally distributed variables are expressed as mean ± standard deviation (SD) and were compared using one-way analysis of variance (ANOVA). When the overall test for LARS was statistically significant, Tukey honestly significant difference (HSD) test was used for prespecified pairwise comparisons. Triglycerides are expressed as median [interquartile range (IQR)] and were compared using the Kruskal–Wallis H test. Categorical variables are expressed as *n* (%) and were compared using the *χ*^2^ test; Fisher exact test or the Fisher–Freeman–Halton exact test was used when expected cell counts were insufficient. Except for the prespecified *post hoc* comparisons for LARS, overall *P* values in the baseline and ABPM tables were used only to describe between-group distributions and not for unplanned pairwise inference. Spearman rank correlation was used to assess monotonic associations between nighttime SBP dipping rate and cardiac structure or left atrial strain, and 95% confidence intervals (CIs) were estimated using Fisher z transformation. The primary exposure was the four-category dipping pattern, with the dipper group as the reference. Continuous dipping rate was used only for supplemental continuous exposure-outcome analyses, and the two exposure representations were not entered into the same model. Continuous LARS was the primary outcome, and low LARS was the operational secondary outcome. The patient-level distribution of the primary outcome was presented using a jittered scatter plot overlaid on a boxplot; the box represented the 25th to 75th percentiles, the horizontal line within the box represented the median, the whiskers represented the minimum and maximum values within 1.5 × IQR, and the horizontal dashed line indicated the prespecified 35% operational threshold. Covariates were determined before model fitting on the basis of clinical prior knowledge, baseline distributions, and presumed pathway factors. Stepwise selection was not used, and univariable *P* values were not used as the sole inclusion criterion. Model 1 was adjusted for age, sex, BMI, and 24-h mean SBP. Model 2 was further adjusted for fasting plasma glucose, natural-log (ln)-transformed triglycerides, low-density lipoprotein cholesterol (LDL-C), high-density lipoprotein cholesterol (HDL-C), and eGFR, and was specified as the primary model. Model 3 further included left ventricular mass index (LVMI), LAVI, and E/e′ as exploratory structural/diastolic-function adjustment. Serum creatinine was not entered into the model simultaneously with eGFR, and total cholesterol was not entered simultaneously with LDL-C and HDL-C. Regression analyses used complete cases, and numerical imputation was not performed. Multicollinearity was assessed using the variance inflation factor (VIF), and VIF <5 was considered to indicate no important multicollinearity. Logistic models reported odds ratios (ORs) and 95% CIs. The 100 low-LARS events corresponded to event-per-parameter ratios of 8.3 and 6.7 for Model 2 and Model 3, respectively; therefore, the low-LARS models were considered secondary analyses, Model 3 was considered exploratory, and Firth penalized-likelihood analysis was used to assess robustness. Continuous dipping rate was analyzed using four-knot restricted cubic spline (RCS) models with knots placed at the 5th, 35th, 65th, and 95th percentiles (−4.5%, 4.9%, 13.6%, and 21.7%); likelihood ratio tests were used to assess overall association and nonlinearity. The linear coefficient per 5-percentage-point increase was interpreted only as a summary of the average association within the observed study range. Sensitivity analyses were also performed among participants with sustained hypertension only, among those with complete sleep diaries only, in models replacing 24-h mean SBP with nighttime SBP, with an alternative threshold, and using Firth models. Intraobserver agreement was assessed with ICC(A,1), and interobserver agreement was assessed with ICC(2,1). All tests were two-sided, with *α* = 0.05.

## 3 Results

### 3.1 Participants and distribution of dipping patterns

Between 1 January 2021 and 31 December 2025, 531 patients were screened. After exclusion of 216 patients, 315 untreated patients with office-defined grade 1 essential hypertension entered the main analysis. There were 126 dippers (40.0%), 124 non-dippers (39.4%), 45 reverse-dippers (14.3%), and 20 extreme-dippers (6.3%); 100 patients (31.7%) had low LARS. The median interval between ABPM and echocardiography was 2 days (IQR 1-4), and 97 patients (30.8%) completed both examinations on the same day. Sleep diaries were used to define awake and asleep periods in 289 patients (91.7%), whereas fixed time windows were used in 26 (8.3%). The sustained-hypertension phenotype was present in 300 patients (95.2%), and the white-coat hypertension phenotype was present in 15 (4.8%) (Figure 1).

### 3.2 Baseline characteristics

Descriptive comparisons showed that the reverse-dipper group had higher age, body weight, fasting plasma glucose, triglycerides, LDL-C, and serum creatinine, and lower HDL-C and eGFR; the overall between-group differences in these variables were statistically significant (all *P* < 0.05). The overall between-group differences in the proportion of men, height, BMI, smoking, alcohol consumption, office SBP, office DBP, heart rate, total cholesterol, and uric acid were not statistically significant (all *P* > 0.05) (Table 1).

**Table 1:** Baseline characteristics stratified by nocturnal blood pressure dipping pattern.

Variable	Overall (*n* = 315)	Dipper (*n* = 126)	Non-dipper (*n* = 124)	Reverse-dipper (*n* = 45)	Extreme-dipper (*n* = 20)	Statistic	*P* value
Age, years	48.8 ± 10.5	46.1 ± 9.5	49.9 ± 10.7	54.3 ± 9.6	46.5 ± 11.5	*F* = 8.23	<0.001
Men, *n* (%)	184 (58.4)	75 (59.5)	70 (56.5)	28 (62.2)	11 (55.0)	*χ*^2^ = 0.63	0.891
Height, cm	167.2 ± 8.2	166.9 ± 8.2	167.3 ± 8.2	169.4 ± 6.9	163.5 ± 9.5	*F* = 2.55	0.056
Weight, kg	72.2 ± 11.8	71.0 ± 11.2	72.6 ± 12.4	76.1 ± 12.1	68.9 ± 8.0	*F* = 2.71	0.045
BMI, kg/m^2^	25.7 ± 3.1	25.4 ± 2.9	25.8 ± 3.3	26.4 ± 3.3	25.7 ± 2.1	*F* = 1.21	0.306
Smoking, *n* (%)	105 (33.3)	38 (30.2)	43 (34.7)	18 (40.0)	6 (30.0)	*χ*^2^ = 1.67	0.643
Alcohol consumption, *n* (%)	92 (29.2)	35 (27.8)	37 (29.8)	15 (33.3)	5 (25.0)	*χ*^2^ = 0.69	0.876
Office SBP, mmHg	148.5 ± 4.4	148.5 ± 4.0	148.9 ± 4.7	147.8 ± 4.5	147.4 ± 4.0	*F* = 1.16	0.326
Office DBP, mmHg	92.2 ± 3.4	91.9 ± 3.4	92.4 ± 3.4	92.9 ± 3.5	91.7 ± 2.5	*F* = 1.27	0.285
Heart rate, beats/min	75.1 ± 8.2	74.0 ± 8.3	75.8 ± 8.3	77.2 ± 8.0	73.1 ± 7.0	*F* = 2.45	0.064
Fasting plasma glucose, mmol/L	5.24 ± 0.51	5.18 ± 0.49	5.20 ± 0.51	5.50 ± 0.52	5.23 ± 0.53	*F* = 4.83	0.003
Total cholesterol, mmol/L	5.17 ± 0.85	5.03 ± 0.89	5.26 ± 0.84	5.35 ± 0.77	5.02 ± 0.72	*F* = 2.51	0.059
Triglycerides, mmol/L	1.56 [1.10, 2.13]	1.49 [1.02, 1.92]	1.62 [1.19, 2.15]	1.94 [1.21, 2.58]	1.33 [0.93, 1.78]	*H* = 9.19	0.027
LDL-C, mmol/L	3.14 ± 0.72	3.02 ± 0.68	3.20 ± 0.73	3.35 ± 0.78	3.01 ± 0.62	*F* = 3.02	0.030
HDL-C, mmol/L	1.26 ± 0.28	1.31 ± 0.27	1.23 ± 0.29	1.18 ± 0.28	1.25 ± 0.25	*F* = 3.06	0.028
Serum creatinine, μmol/L	73.8 ± 13.7	73.0 ± 12.8	73.4 ± 14.9	79.0 ± 12.7	70.4 ± 12.2	*F* = 2.78	0.041
eGFR, mL/min/1.73 m^2^	100.6 ± 10.2	102.9 ± 9.9	99.9 ± 9.9	96.2 ± 10.1	101.0 ± 11.1	*F* = 5.33	0.001
Uric acid, μmol/L	358.5 ± 84.8	355.7 ± 84.1	363.1 ± 80.1	371.0 ± 101.9	319.0 ± 68.2	*F* = 1.96	0.121

Approximately normally distributed continuous variables are shown as mean ± standard deviation (SD), triglycerides as median [interquartile range (IQR)], and categorical variables as *n* (%). Overall *P* values were obtained using one-way analysis of variance (ANOVA), Kruskal–Wallis H test, Pearson *χ*^2^ test, or Fisher exact test as appropriate. Overall comparisons were used only to describe between-group distributions, and unplanned pairwise comparisons were not interpreted. *F*, *F* statistic from ANOVA; *H*, Kruskal–Wallis *H* statistic; *χ*^2^, Pearson chi-square statistic; *P*, two-sided *P* value; *n*, number of participants; %, percentage; Fisher exact test, an exact-probability method for comparing categorical variables; BMI, body mass index; DBP, diastolic blood pressure; eGFR, estimated glomerular filtration rate; HDL-C, high-density lipoprotein cholesterol; LDL-C, low-density lipoprotein cholesterol; SBP, systolic blood pressure.

### 3.3 Ambulatory blood pressure monitoring

The reverse-dipper group had the highest 24-h mean SBP, 24-h mean DBP, nighttime mean SBP, and nighttime mean DBP, whereas the extreme-dipper group had the lowest values. The nighttime DBP dipping rate was lowest in the reverse-dipper group and highest in the extreme-dipper group, and the proportion with sustained hypertension was lower in the extreme-dipper group. The overall between-group differences in these variables were statistically significant (all *P* < 0.05). Monitoring duration, valid reading proportion, number of valid readings, daytime mean SBP, and daytime mean DBP did not differ significantly across groups (all *P* > 0.05). Nighttime SBP dipping rate was the grouping variable and is presented descriptively only (Table 2).

**Table 2:** Twenty-four-hour ambulatory blood pressure monitoring results stratified by nocturnal blood pressure dipping pattern.

Variable	Overall (*n* = 315)	Dipper (*n* = 126)	Non-dipper (*n* = 124)	Reverse-dipper (*n* = 45)	Extreme-dipper (*n* = 20)	Statistic	*P* value
Monitoring duration, h	24.7 ± 0.4	24.7 ± 0.3	24.7 ± 0.4	24.7 ± 0.4	24.9 ± 0.4	*F* = 1.89	0.131
Valid reading proportion, %	90.8 ± 4.5	91.1 ± 4.7	90.8 ± 4.5	89.6 ± 4.2	91.8 ± 3.8	*F* = 1.58	0.194
Number of valid readings	61.1 ± 6.2	61.4 ± 6.5	61.4 ± 6.1	59.6 ± 5.6	61.0 ± 5.8	*F* = 1.08	0.357
24-h mean SBP, mmHg	133.6 ± 6.9	131.5 ± 5.9	135.4 ± 6.7	137.4 ± 6.5	126.8 ± 5.3	*F* = 21.40	<0.001
24-h mean DBP, mmHg	84.2 ± 5.7	83.4 ± 4.8	85.0 ± 6.0	86.5 ± 5.7	78.4 ± 4.9	*F* = 12.07	<0.001
Daytime mean SBP, mmHg	137.7 ± 6.5	138.3 ± 6.2	137.8 ± 7.0	135.8 ± 6.0	137.5 ± 5.3	*F* = 1.68	0.171
Daytime mean DBP, mmHg	86.7 ± 5.5	87.3 ± 4.9	86.7 ± 6.0	86.1 ± 5.6	84.8 ± 5.0	*F* = 1.49	0.217
Nighttime mean SBP, mmHg	125.6 ± 11.7	118.0 ± 5.9	130.9 ± 6.7	141.0 ± 7.8	105.4 ± 5.5	*F* = 233.75	<0.001
Nighttime mean DBP, mmHg	78.8 ± 8.0	75.3 ± 5.1	81.4 ± 6.7	87.0 ± 6.8	66.0 ± 6.0	*F* = 78.34	<0.001
Nighttime SBP dipping rate, %	8.7 ± 7.9	14.6 ± 2.4	4.9 ± 2.4	−3.9 ± 2.4	23.4 ± 2.3	Grouping criterion	—
Nighttime DBP dipping rate, %	9.2 ± 7.4	13.8 ± 3.5	6.1 ± 4.3	−1.0 ± 4.5	22.3 ± 4.8	*F* = 241.72	<0.001
Sustained-hypertension phenotype, *n* (%)	300 (95.2)	119 (94.4)	121 (97.6)	44 (97.8)	16 (80.0)	Fisher–Freeman–Halton	0.020

Continuous variables are shown as mean ± standard deviation (SD), and overall between-group comparisons used one-way analysis of variance (ANOVA). The proportion with a sustained-hypertension phenotype was compared using the Fisher–Freeman–Halton exact test. Nighttime systolic blood pressure (SBP) dipping rate was the defining variable for the four groups and is presented descriptively only, without between-group inferential testing. A valid ambulatory blood pressure monitoring (ABPM) record required an overall valid reading proportion ≥70%, ≥20 awake readings, ≥7 asleep readings, and no interval >2 h without a valid reading. *F*, *F* statistic from ANOVA; *n*, number of participants; *P*, two-sided *P* value; %, percentage; —, no inferential test performed; Fisher–Freeman–Halton exact test, an exact-probability test for multicategory contingency tables; ABPM, 24-h ambulatory blood pressure monitoring; BP, blood pressure; DBP, diastolic blood pressure; SBP, systolic blood pressure.

### 3.4 Echocardiography and strain

**Figure 2:**
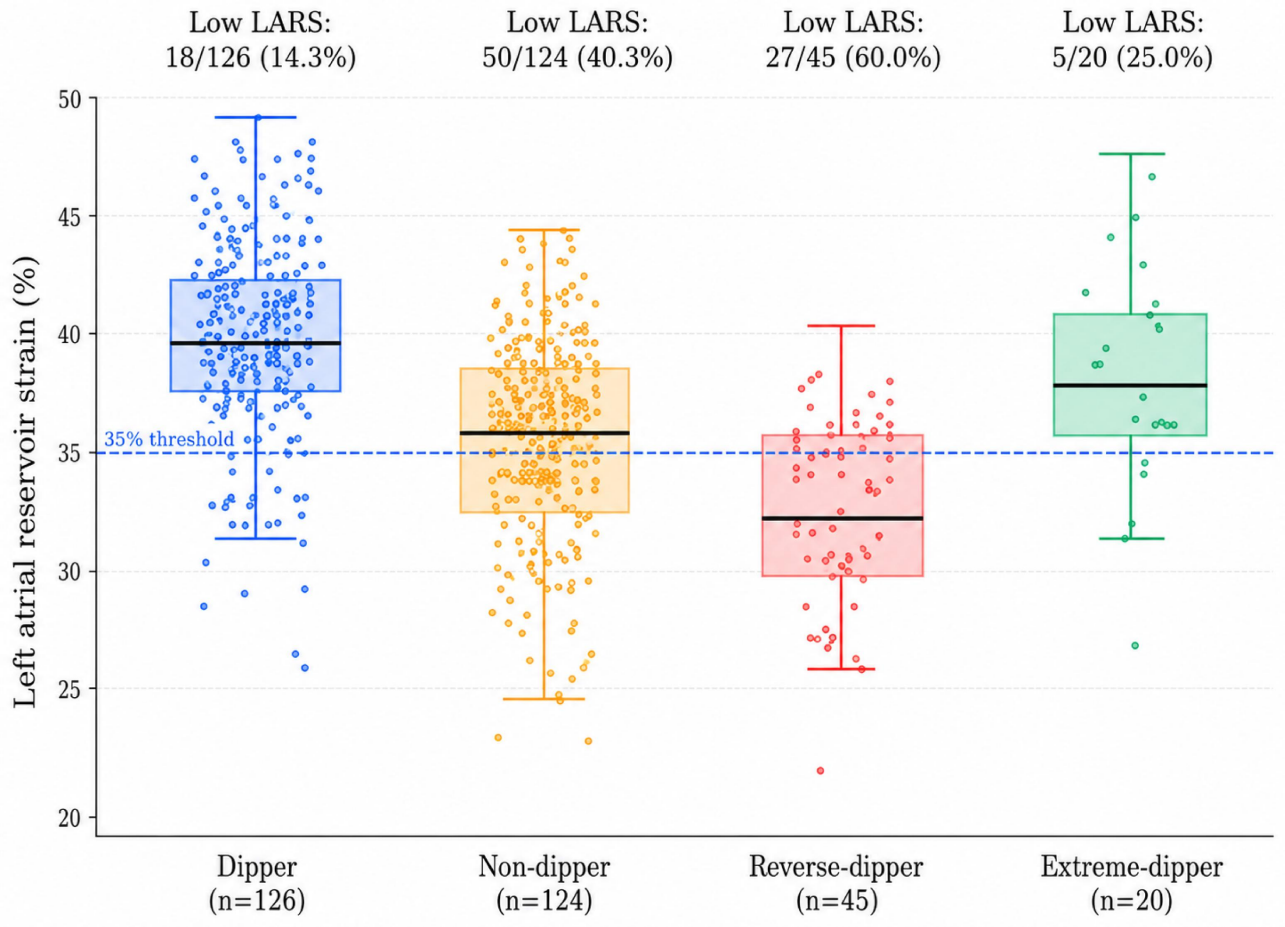
Patient-level distribution of left atrial reservoir strain (LARS) by nocturnal blood pressure dipping pattern. Each point represents the LARS value of 1 patient (*n* = 315); the box represents the 25th to 75th percentiles, the horizontal line within the box represents the median, and the whiskers represent the minimum and maximum values within 1.5 × IQR. The horizontal dashed line indicates the prespecified 35% operational threshold; text annotations show the number/total number and proportion of patients with low LARS (<35%) in each group. SBP, systolic blood pressure; *n*, number of participants; IQR, interquartile range; LARS, left atrial reservoir strain.

The reverse-dipper group had higher left ventricular posterior wall thickness, LVMI, LAVI, and proportion of low LARS, and lower mitral E-wave velocity, mitral annular e′ velocity, LARS, left atrial conduit strain, and left atrial contraction strain. E/e′ was higher in the non-dipper and reverse-dipper groups. The overall between-group differences in these variables were statistically significant (all *P* < 0.05). The overall between-group differences in left ventricular end-diastolic diameter, interventricular septal thickness, and LVEF were not statistically significant (all *P* > 0.05). *Post hoc* comparisons of LARS showed that the dipper group had higher LARS than the non-dipper and reverse-dipper groups, the non-dipper group had higher LARS than the reverse-dipper group, and the reverse-dipper group had lower LARS than the extreme-dipper group (all adjusted *P* < 0.05). Differences between the dipper and extreme-dipper groups and between the non-dipper and extreme-dipper groups were not statistically significant (all adjusted *P* > 0.05) (Table 3, Figure 2, Supplementary Table S1).

**Table 3:** Echocardiographic and left atrial strain variables stratified by nocturnal blood pressure dipping pattern.

Variable	Overall (*n* = 315)	Dipper (*n* = 126)	Non-dipper (*n* = 124)	Reverse-dipper (*n* = 45)	Extreme-dipper (*n* = 20)	Statistic	*P* value
Left ventricular end-diastolic diameter, mm	47.6 ± 3.3	47.3 ± 3.3	47.5 ± 3.5	48.1 ± 2.8	48.5 ± 3.5	*F* = 1.20	0.312
Interventricular septal thickness, mm	9.7 ± 1.0	9.6 ± 1.0	9.8 ± 1.0	10.0 ± 1.1	9.5 ± 0.6	*F* = 2.38	0.070
Left ventricular posterior wall thickness, mm	9.6 ± 1.0	9.4 ± 0.9	9.7 ± 1.1	10.0 ± 1.0	9.5 ± 1.0	*F* = 4.52	0.004
LVEF, %	64.2 ± 4.2	64.8 ± 3.8	63.6 ± 4.2	64.4 ± 4.8	64.0 ± 4.3	*F* = 1.80	0.148
Mitral E-wave velocity, cm/s	71.0 ± 12.9	72.8 ± 14.1	71.3 ± 12.3	66.3 ± 9.8	68.8 ± 12.8	*F* = 3.08	0.028
Mitral annular e′ velocity, cm/s	9.9 ± 2.1	10.6 ± 2.2	9.5 ± 1.8	8.9 ± 1.6	10.4 ± 2.2	*F* = 11.29	<0.001
E/e′	7.5 ± 2.2	7.2 ± 2.2	7.9 ± 2.2	7.7 ± 1.9	7.0 ± 1.9	*F* = 2.73	0.044
LVMI, g/m^2^	86.5 ± 16.9	81.9 ± 14.9	87.8 ± 17.7	95.8 ± 16.9	86.5 ± 14.7	*F* = 8.46	<0.001
LAVI, mL/m^2^	26.5 ± 5.7	24.4 ± 4.7	27.6 ± 5.8	29.5 ± 6.0	26.6 ± 5.6	*F* = 12.68	<0.001
LARS, %	36.9 ± 5.0	39.5 ± 4.4	35.6 ± 4.5	32.8 ± 3.9	37.8 ± 4.7	*F* = 31.69	<0.001
Left atrial conduit strain, %	21.2 ± 3.8	22.7 ± 3.5	20.6 ± 3.6	18.5 ± 3.3	21.5 ± 2.9	*F* = 18.12	<0.001
Left atrial contraction strain, %	15.8 ± 3.4	16.9 ± 3.4	15.3 ± 3.2	14.3 ± 3.0	16.1 ± 3.3	*F* = 8.96	<0.001
Low LARS (<35%), *n* (%)	100 (31.7)	18 (14.3)	50 (40.3)	27 (60.0)	5 (25.0)	*χ*^2^ = 38.94	<0.001

Continuous variables are shown as mean ± standard deviation (SD), and low LARS is shown as *n* (%). Overall comparisons used one-way analysis of variance (ANOVA) or the Pearson *χ*^2^ test. LARS was the prespecified primary continuous outcome; low LARS (<35%) was the operational secondary outcome. After the overall ANOVA for LARS was statistically significant, Tukey HSD testing was used for prespecified pairwise comparisons; adjusted results are listed in Supplementary Table S1. *F*, *F* statistic from ANOVA; *χ*^2^, Pearson chi-square statistic; *P*, two-sided *P* value; *n*, number of participants; %, percentage; HSD, honestly significant difference, a multiple-comparison adjustment method; E/e′, ratio of mitral early diastolic E-wave velocity to mitral annular early diastolic e′ velocity; LARS, left atrial reservoir strain; LAVI, left atrial volume index; LVEF, left ventricular ejection fraction; LVMI, left ventricular mass index.

Patient-level distributions indicated that LARS values in the non-dipper and reverse-dipper groups were shifted overall toward lower values; the proportions with low LARS (<35%) were 50/124 (40.3%) and 27/45 (60.0%), respectively (Figure 2).

### 3.5 Correlations

Nighttime SBP dipping rate was positively correlated with LARS, left atrial conduit strain, and left atrial contraction strain, and negatively correlated with LAVI, E/e′, and LVMI (all *P* < 0.05) (Supplementary Table S2).

### 3.6 Regression analyses and sensitivity analyses

**Figure 3:**
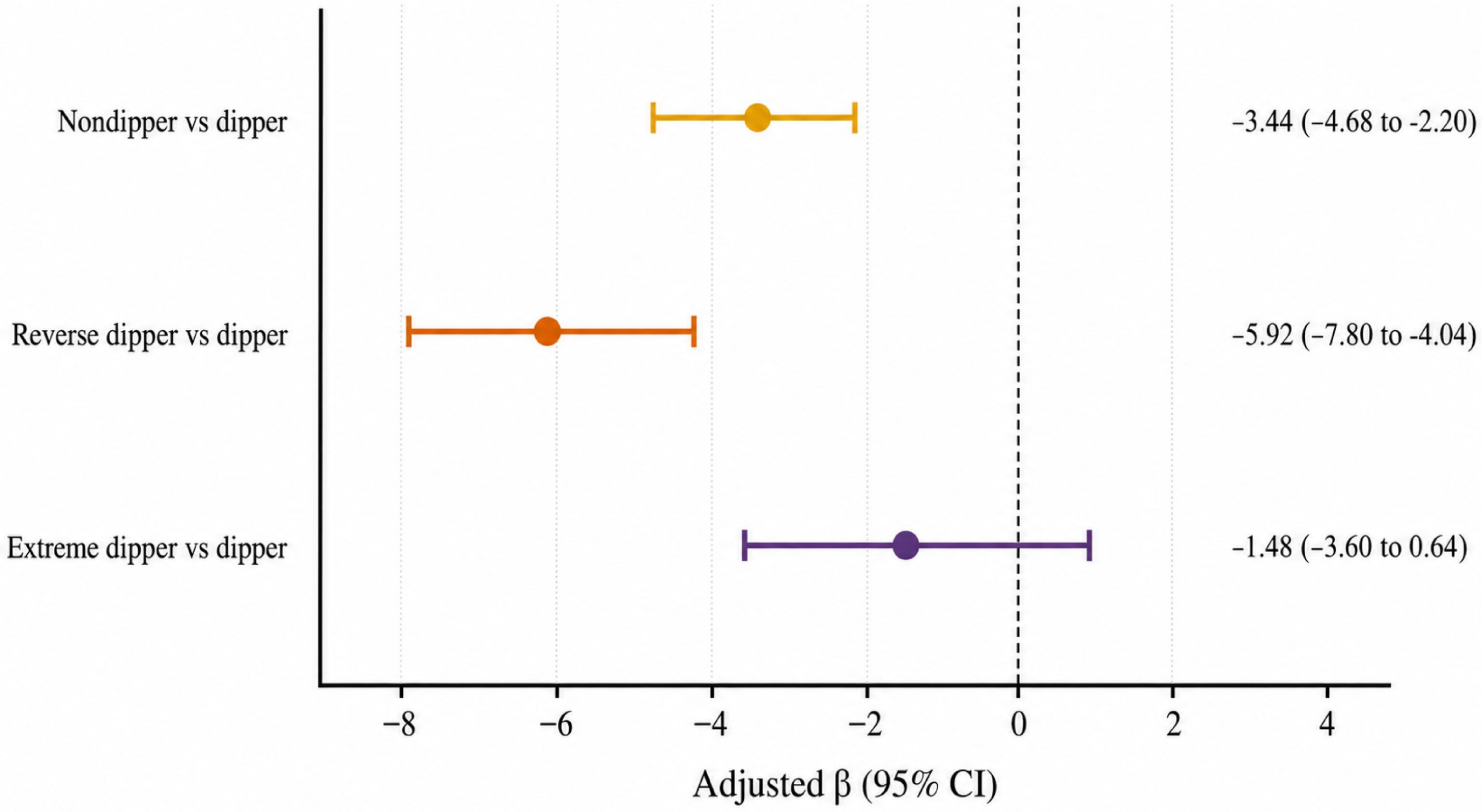
Association between nocturnal blood pressure dipping pattern and continuous left atrial reservoir strain (LARS). Dots represent Model 2-adjusted unstandardized linear regression coefficients (*β*), horizontal lines represent 95% confidence intervals (CIs), and the vertical dashed line represents the null value of 0; the dipper group is the reference. Model 2 was adjusted for age, sex, body mass index (BMI), 24-h mean systolic blood pressure (SBP), fasting plasma glucose, natural-log (ln)-transformed triglycerides, low-density lipoprotein cholesterol (LDL-C), high-density lipoprotein cholesterol (HDL-C), and estimated glomerular filtration rate (eGFR). vs., versus.

**Figure 4:**
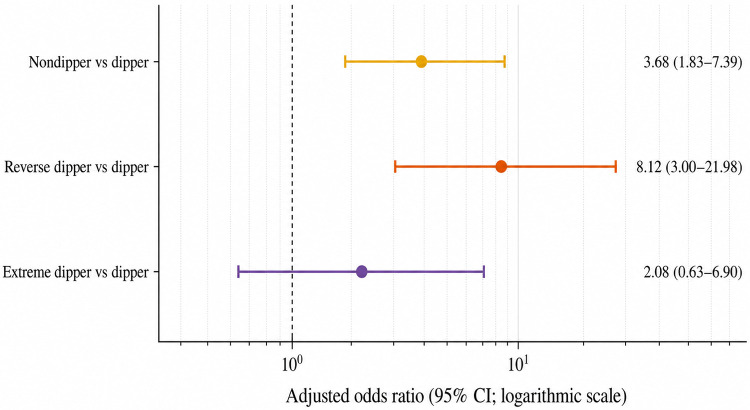
Association between nocturnal blood pressure dipping pattern and low left atrial reservoir strain (LARS; <35%). Dots represent Model 2-adjusted odds ratios (ORs), horizontal lines represent 95% confidence intervals (CIs), and the vertical dashed line represents the null value of 1. The *x*-axis uses a logarithmic scale, and the dipper group is the reference. Model 2 was adjusted for age, sex, body mass index (BMI), 24-h mean systolic blood pressure (SBP), fasting plasma glucose, natural-log (ln)-transformed triglycerides, low-density lipoprotein cholesterol (LDL-C), high-density lipoprotein cholesterol (HDL-C), and estimated glomerular filtration rate (eGFR). vs., versus.

In Models 1–3, non-dipper and reverse-dipper BP dipping patterns were associated with lower LARS and a higher likelihood of low LARS (all *P* < 0.05), whereas extreme-dipping was not statistically significant in any model (all *P* > 0.05). Model 2 was the primary model; after LVMI, LAVI, and E/e′, which may lie on the causal pathway, were added, effect estimates in Model 3 were attenuated (Tables 4, 5, Figures 3, 4).

**Table 4:** Multivariable linear regression of nocturnal blood pressure dipping patterns with continuous left atrial reservoir strain (LARS).

Contrast	Model	*β*	95% CI	*t*	*P* value
Non-dipper vs. dipper	Model 1	−3.78	−5.00 to −2.56	−6.07	<0.001
Reverse-dipper vs. dipper	Model 1	−6.54	−8.36 to −4.72	−7.04	<0.001
Extreme-dipper vs. dipper	Model 1	−1.61	−3.74 to 0.52	−1.48	0.140
Non-dipper vs. dipper	Model 2 (primary)	−3.44	−4.68 to −2.20	−5.44	<0.001
Reverse-dipper vs. dipper	Model 2 (primary)	−5.92	−7.80 to −4.04	−6.17	<0.001
Extreme-dipper vs. dipper	Model 2 (primary)	−1.48	−3.60 to 0.64	−1.37	0.172
Non-dipper vs. dipper	Model 3 (exploratory)	−2.98	−4.18 to −1.78	−4.87	<0.001
Reverse-dipper vs. dipper	Model 3 (exploratory)	−5.06	−6.88 to −3.24	−5.45	<0.001
Extreme-dipper vs. dipper	Model 3 (exploratory)	−1.43	−3.45 to 0.59	−1.39	0.166

*β* denotes the unstandardized multivariable linear regression coefficient, *t* denotes the *t* statistic for the coefficient test, CI denotes confidence interval, and P denotes a two-sided *P* value. vs., comparison of the former group with the latter group; 95% CI, 95% confidence interval. Model 1 was adjusted for age, sex, BMI, and 24-h mean SBP. Model 2 was further adjusted for fasting plasma glucose, natural-log (ln)-transformed triglycerides, low-density lipoprotein cholesterol (LDL-C), high-density lipoprotein cholesterol (HDL-C), and eGFR, and was specified as the primary model. Model 3 further included LVMI, LAVI, and E/e′ as exploratory structural/diastolic-function adjustment. Regression analyses used complete cases, without numerical imputation. The maximum VIFs for Model 1, Model 2, and Model 3 were 1.54, 2.08, and 2.31, respectively. BMI, body mass index; eGFR, estimated glomerular filtration rate; HDL-C, high-density lipoprotein cholesterol; LARS, left atrial reservoir strain; LAVI, left atrial volume index; LDL-C, low-density lipoprotein cholesterol; LVMI, left ventricular mass index; SBP, systolic blood pressure; VIF, variance inflation factor.

**Table 5:** Multivariable logistic regression of nocturnal blood pressure dipping patterns with low left atrial reservoir strain (LARS).

Contrast	Model	OR	95% CI	Wald *χ*^2^	*P* value
Non-dipper vs. dipper	Model 1	4.20	2.15–8.21	17.63	<0.001
Reverse-dipper vs. dipper	Model 1	9.70	3.88–24.24	23.63	<0.001
Extreme-dipper vs. dipper	Model 1	2.35	0.75–7.34	2.16	0.142
Non-dipper vs. dipper	Model 2 (primary)	3.68	1.83–7.39	13.39	<0.001
Reverse-dipper vs. dipper	Model 2 (primary)	8.12	3.00–21.98	16.99	<0.001
Extreme-dipper vs. dipper	Model 2 (primary)	2.08	0.63–6.90	1.44	0.230
Non-dipper vs. dipper	Model 3 (exploratory)	3.21	1.57–6.56	10.22	0.001
Reverse-dipper vs. dipper	Model 3 (exploratory)	6.63	2.42–18.18	13.52	<0.001
Extreme-dipper vs. dipper	Model 3 (exploratory)	1.96	0.58–6.62	1.17	0.279

OR denotes the odds ratio from multivariable logistic regression, Wald *χ*^2^ denotes the Wald chi-square statistic for the regression coefficient, CI denotes confidence interval, and P denotes a two-sided *P* value. vs., comparison of the former group with the latter group; 95% CI, 95% confidence interval. Model 1 was adjusted for age, sex, BMI, and 24-h mean SBP. Model 2 was further adjusted for fasting plasma glucose, natural-log (ln)-transformed triglycerides, low-density lipoprotein cholesterol (LDL-C), high-density lipoprotein cholesterol (HDL-C), and eGFR, and was specified as the primary model. Model 3 further included LVMI, LAVI, and E/e′ as exploratory structural/diastolic-function adjustment. Regression analyses used complete cases, without numerical imputation. The maximum VIFs for Model 1, Model 2, and Model 3 were 1.54, 2.08, and 2.31, respectively. BMI, body mass index; eGFR, estimated glomerular filtration rate; HDL-C, high-density lipoprotein cholesterol; LARS, left atrial reservoir strain; LAVI, left atrial volume index; LDL-C, low-density lipoprotein cholesterol; LVMI, left ventricular mass index; SBP, systolic blood pressure; VIF, variance inflation factor.

Continuous dipping rate was associated with higher LARS and a lower likelihood of low LARS (all *P* < 0.05). The VIFs for this variable in Models 1–3 were 1.24, 1.31, and 1.38, respectively. RCS analyses suggested nonlinearity for both continuous LARS and low LARS (nonlinear *P* = 0.018 and *P* = 0.043); therefore, the linear estimate per 5-percentage-point increase represented only the average association within the study range (Supplementary Table S3). Effect directions were consistent with the main analysis in the alternative-threshold, complete-sleep-diary, sustained-hypertension phenotype, and Firth analyses. When nighttime SBP replaced 24-h mean SBP, the effects for non-dipping and reverse-dipping were attenuated but remained statistically significant, whereas the effect for extreme-dipping remained nonsignificant.

All reported VIFs in the main and sensitivity models were <5: the maximum VIFs in categorical exposure Models 1, 2, and 3 were 1.54, 2.08, and 2.31, respectively; the maximum VIF in the continuous dipping-rate models was 1.38; and the maximum VIF in the sensitivity model replacing 24-h mean SBP with nighttime SBP was 3.42, suggesting no important multicollinearity (Tables 4, 5, Supplementary Tables S3, S7).

### 3.7 Reproducibility

Intraobserver and interobserver agreement for the three left atrial strain parameters was good to excellent (all *P* < 0.001) (Supplementary Table S9).

## 4 Discussion

This study showed that, among untreated patients with office-defined grade 1 essential hypertension, non-dipper and reverse-dipper nocturnal BP patterns were associated with lower LARS and a higher likelihood of low LARS; extreme-dipping was not statistically associated with either outcome. Recent evidence indicates that nighttime BP and insufficient nocturnal BP dipping may have stronger associations with outcomes such as heart failure than office BP, consistent with existing evidence that ABPM phenotyping helps characterize hypertension-related nighttime load (21). When the analysis was restricted to 300 patients with a sustained-hypertension phenotype, the direction of the main associations remained consistent, indicating that the 15 patients with a white-coat hypertension phenotype did not drive the main estimates.

LARS reflects left atrial compliance and reservoir function during left ventricular systole and may reveal functional alterations before overt left atrial enlargement (22). LARS is sensitive to loading changes in a range of cardiovascular and systemic diseases (23). In the present study, the reverse-dipper group had higher LVMI and LAVI and lower e′ velocity and all three left atrial strain parameters, consistent with the framework of hypertensive heart disease progressing from pressure load to structural and functional remodeling (24). When changes in conventional echocardiographic parameters remain relatively mild, 2D-STE may reveal hypertension-related cardiac functional differences (25).

After adjustment for fasting plasma glucose, lipids, and eGFR, the main associations remained statistically significant, suggesting that differences in cardiometabolic and renal function factors did not fully explain the observed associations within the range of measured covariates. A recent framework for assessing filling pressure has incorporated LARS into the estimation of left ventricular filling pressure, suggesting that LARS may provide complementary information related to diastolic load (26). OSA-related hypertension commonly manifests as elevated nighttime BP and abnormal dipping patterns (27) and is associated with arrhythmia and abnormal autonomic regulation (28). Sympathetic activation, intermittent hypoxia, and BP variability may jointly contribute to this relationship (29). Sleep quality and nighttime BP management provide important context for interpreting hypertension phenotypes (30), and current evidence also emphasizes the importance of systematic OSA screening in nocturnal BP research (31).

When nighttime SBP replaced 24-h mean SBP, the effects for non-dipping and reverse-dipping were substantially attenuated but remained statistically significant, suggesting that absolute nighttime pressure load may explain part of the observed association. The residual association should still be interpreted cautiously in light of the cross-sectional design, a single ABPM measurement, and the mathematical construction of the dipping rate. Because the dipping rate is calculated from daytime and nighttime SBP, cross-sectional data have limited ability to distinguish the independent causal contributions of dipping pattern and nighttime SBP. Sleep posture and the height difference between the cuff and the heart can alter nighttime readings and dipping classification (32); therefore, single-session ABPM phenotyping may contain measurement variability. Nighttime hypertension and dipping status should be interpreted together with patient characteristics and absolute nighttime BP (33). ABPM phenotypes and implementation conditions also vary regionally across Asian populations (34).

The RCS test for continuous dipping rate suggested nonlinearity, and the four-category results did not show that a greater BP dip was necessarily associated with higher LARS. Therefore, the linear coefficient per 5-percentage-point increase should be interpreted only as a summary of the average association within the study range, and clinical interpretation should remain centered on the four-category dipping patterns. Patients with hypertension may have reduced strain even when left atrial size remains normal (35). Reference data from healthy populations suggest a mean LARS of approximately 39%, but between-study heterogeneity and vendor differences exist (36). Therefore, this study used continuous LARS as the primary outcome and regarded 35% as an operational secondary cutoff.

This study has several limitations. First, the single-center retrospective cross-sectional design limited the assessment of temporal sequence, causal inference, and external generalizability. Second, BP dipping patterns were based on a single ABPM recording. Although sensitivity analyses restricted to participants with complete sleep diaries yielded consistent findings, self-reported sleep time, body position, and single-recording variability may still have caused misclassification. Third, the use of the same vendor and software for all image analyses improved internal consistency, but differences in vendor, software version, reference zero point, and tracking algorithm may limit the transferability of absolute LARS values and cutoffs. Fourth, the number of binary low-LARS events and the number of events in the extreme-dipper group were limited, resulting in wide confidence intervals for the corresponding ORs. Continuous LARS was the primary outcome, low LARS and Model 3 were secondary and exploratory analyses, respectively, and Firth and alternative-threshold analyses were used to assess robustness. Fifth, this study excluded only patients with moderate-to-severe OSA previously diagnosed by sleep-specialist evaluation or polysomnography and did not systematically screen all patients. Undiagnosed OSA may be associated with higher nighttime BP, non-dipper or reverse-dipper patterns, and left atrial remodeling simultaneously, thereby biasing the estimated association between non-dipping or reverse-dipping and lower LARS away from the null and overestimating the effect size. BMI data were available for all participants and included in the primary model, but BMI is a limited proxy for OSA assessment; the Epworth Sleepiness Scale and nocturnal oxygen saturation were not systematically recorded. Sixth, LVMI, LAVI, and E/e′ may lie on the pathway between the exposure and LARS; Model 3 was used to explore the degree of attenuation in effect estimates, and formal mediation quantification requires a dedicated design and longitudinal data. Seventh, this study did not include long-term cardiovascular outcomes, incremental prediction, or external validation.

## 5 Conclusions

This single-center retrospective cross-sectional study showed that, among untreated patients with office-defined grade 1 essential hypertension, non-dipper and reverse-dipper nocturnal BP patterns were associated with lower LARS and a higher likelihood of low LARS (<35%). The association persisted after adjustment for 24-h mean SBP, cardiometabolic factors, and renal function factors, and was attenuated after further consideration of nighttime SBP load and structural/diastolic-function variables. These findings indicate only cross-sectional associations between nocturnal BP rhythm and left atrial function. The prognostic value and the incremental clinical value of combining ABPM with left atrial strain require validation in prospective multicenter studies.

## Data Availability

The original contributions presented in the study are included in the article/Supplementary Material, further inquiries can be directed to the corresponding authors.
