# Supplementary material for: Association between nocturnal blood pressure dipping patterns and left atrial reservoir strain in untreated adults with office-defined grade 1 essential hypertension
Source: Front Cardiovasc Med. 2026 Jul 27;13:1898739. doi: 10.3389/fcvm.2026.1898739 (PMC13453675; doi:10.3389/fcvm.2026.1898739)
Supplement: Supplementary file 1 [file Table1.docx]

# Supplementary Materials

**Supplementary Table 1. Tukey HSD post hoc comparisons for left atrial reservoir strain (LARS)**

| **Group comparison** | **Mean difference** | **95% CI** | **Adjusted *P* value** |
| --- | --- | --- | --- |
| Dipper vs non-dipper | 3.90 | 2.46-5.34 | <0.001 |
| Dipper vs reverse-dipper | 6.70 | 4.73-8.67 | <0.001 |
| Dipper vs extreme-dipper | 1.70 | -1.03 to 4.43 | 0.376 |
| Non-dipper vs reverse-dipper | 2.80 | 0.83-4.77 | 0.002 |
| Non-dipper vs extreme-dipper | -2.20 | -4.93 to 0.53 | 0.162 |
| Reverse-dipper vs extreme-dipper | -5.00 | -8.05 to -1.95 | <0.001 |

Note: The prespecified Tukey HSD pairwise comparisons were performed only for the primary continuous outcome, LARS. Mean difference was calculated as the former group minus the latter group. CI, confidence interval; LARS, left atrial reservoir strain; *P*, two-sided *P* value adjusted for multiple comparisons using HSD; HSD, honestly significant difference; vs, comparison of the former group with the latter group; 95% CI, 95% confidence interval.

**Supplementary Table 2. Correlations between nighttime systolic blood pressure (SBP) dipping rate and cardiac structure and left atrial strain**

| **Variable** | **Spearman *ρ*** | **95% CI** | ***P* value** |
| --- | --- | --- | --- |
| LARS, % | 0.425 | 0.329-0.512 | <0.001 |
| Left atrial conduit strain, % | 0.337 | 0.234-0.433 | <0.001 |
| Left atrial contraction strain, % | 0.222 | 0.113-0.326 | <0.001 |
| LAVI, mL/m² | -0.280 | -0.381 to -0.173 | <0.001 |
| E/e′ | -0.132 | -0.240 to -0.021 | 0.019 |
| LVMI, g/m² | -0.218 | -0.322 to -0.109 | <0.001 |

Note: Spearman rank correlation was used. *ρ* denotes the Spearman rank correlation coefficient, 95% CI was estimated using Fisher z transformation, and P denotes a two-sided *P* value. 95% CI, 95% confidence interval; E/e′, ratio of mitral early diastolic E-wave velocity to mitral annular early diastolic e′ velocity; LARS, left atrial reservoir strain; LAVI, left atrial volume index; LVMI, left ventricular mass index; SBP, systolic blood pressure.

**Supplementary Table 3. Continuous analysis of nighttime systolic blood pressure (SBP) dipping rate**

| **Outcome** | **Continuous exposure** | **Model** | **Effect** | **95% CI** | ***P* value** | **Dipping-rate VIF** |
| --- | --- | --- | --- | --- | --- | --- |
| LARS | Per 5-percentage-point increase | Model 1 | *β*=1.21 | 0.84-1.58 | <0.001 | 1.24 |
| LARS | Per 5-percentage-point increase | Model 2 | *β*=1.12 | 0.75-1.49 | <0.001 | 1.31 |
| LARS | Per 5-percentage-point increase | Model 3 | *β*=0.95 | 0.59-1.31 | <0.001 | 1.38 |
| Low LARS | Per 5-percentage-point increase | Model 1 | OR=0.65 | 0.53-0.80 | <0.001 | 1.24 |
| Low LARS | Per 5-percentage-point increase | Model 2 | OR=0.67 | 0.54-0.83 | <0.001 | 1.31 |
| Low LARS | Per 5-percentage-point increase | Model 3 | OR=0.72 | 0.58-0.89 | 0.003 | 1.38 |
| LARS | RCS (4 knots) | Model 2 | Overall association | — | Overall <0.001; nonlinear=0.018 | — |
| Low LARS | RCS (4 knots) | Model 2 | Overall association | — | Overall <0.001; nonlinear=0.043 | — |

Note: Categorical dipping pattern and continuous dipping rate were modeled separately. *β* denotes the linear regression coefficient, OR denotes the odds ratio from logistic regression, CI denotes confidence interval, P denotes a two-sided *P* value, and VIF denotes variance inflation factor. The covariates in Models 1, 2, and 3 were the same as those in Tables 4 and 5. Coefficients per 5-percentage-point increase represent only the average association within the study range. Restricted cubic spline (RCS) tests were used to assess overall association and nonlinearity. 95% CI, 95% confidence interval; LARS, left atrial reservoir strain; SBP, systolic blood pressure.

**Supplementary Table 4. Firth logistic analysis using left atrial reservoir strain (LARS) <30% as an alternative threshold**

| **Group** | **LARS <30%, n/N (%)** | **Adjusted OR** | **95% CI** | ***P* value** |
| --- | --- | --- | --- | --- |
| Dipper | 2/126 (1.6) | Reference | — | — |
| Non-dipper | 13/124 (10.5) | 5.08 | 1.21-21.30 | 0.026 |
| Reverse-dipper | 11/45 (24.4) | 10.92 | 2.36-50.59 | 0.002 |
| Extreme-dipper | 1/20 (5.0) | 2.47 | 0.29-21.13 | 0.409 |

Note: Firth penalized-likelihood logistic regression was used, with LARS <30% as the outcome and covariates identical to those in Model 2. n/N, number with outcome/total number in the group; CI, confidence interval; LARS, left atrial reservoir strain; OR, odds ratio; *P*, two-sided *P* value; %, percentage; 95% CI, 95% confidence interval; Firth penalized-likelihood method, a method used to reduce estimation bias in small-sample or rare-event logistic regression.

**Supplementary Table 5. Sensitivity analysis restricted to patients with a sustained-hypertension phenotype (n=300)**

| **Outcome** | **Contrast** | **Effect estimate** | **95% CI** | ***P* value** |
| --- | --- | --- | --- | --- |
| LARS | Non-dipper vs dipper | *β*=-3.38 | -4.65 to -2.11 | <0.001 |
| LARS | Reverse-dipper vs dipper | *β*=-5.82 | -7.73 to -3.91 | <0.001 |
| LARS | Extreme-dipper vs dipper | *β*=-1.35 | -3.66 to 0.96 | 0.253 |
| Low LARS | Non-dipper vs dipper | OR=3.61 | 1.78-7.34 | <0.001 |
| Low LARS | Reverse-dipper vs dipper | OR=7.96 | 2.90-21.85 | <0.001 |
| Low LARS | Extreme-dipper vs dipper | OR=1.89 | 0.52-6.90 | 0.334 |

Note: The analysis included only the 300 patients who met criteria for a sustained-hypertension phenotype. Model covariates were identical to those in Model 2. *β* denotes the linear regression coefficient, OR denotes the odds ratio from logistic regression, CI denotes confidence interval, and P denotes a two-sided *P* value. LARS, left atrial reservoir strain; vs, comparison of the former group with the latter group; 95% CI, 95% confidence interval.

**Supplementary Table 6. Sensitivity analysis restricted to patients with complete sleep diaries (n=289)**

| **Outcome** | **Contrast** | **Effect estimate** | **95% CI** | ***P* value** |
| --- | --- | --- | --- | --- |
| LARS | Non-dipper vs dipper | *β*=-3.39 | -4.67 to -2.11 | <0.001 |
| LARS | Reverse-dipper vs dipper | *β*=-5.81 | -7.77 to -3.85 | <0.001 |
| LARS | Extreme-dipper vs dipper | *β*=-1.41 | -3.64 to 0.82 | 0.216 |
| Low LARS | Non-dipper vs dipper | OR=3.61 | 1.74-7.49 | <0.001 |
| Low LARS | Reverse-dipper vs dipper | OR=7.74 | 2.77-21.62 | <0.001 |
| Low LARS | Extreme-dipper vs dipper | OR=1.92 | 0.53-6.96 | 0.321 |

Note: The analysis included only the 289 patients whose awake and asleep periods were defined using complete sleep diaries. Model covariates were identical to those in Model 2. *β* denotes the linear regression coefficient, OR denotes the odds ratio from logistic regression, CI denotes confidence interval, and P denotes a two-sided *P* value. LARS, left atrial reservoir strain; vs, comparison of the former group with the latter group; 95% CI, 95% confidence interval.

**Supplementary Table 7. Sensitivity analysis replacing 24-hour mean systolic blood pressure (SBP) with nighttime mean SBP**

| **Outcome** | **Contrast** | **Effect estimate** | **95% CI** | ***P* value** |
| --- | --- | --- | --- | --- |
| LARS | Non-dipper vs dipper | *β*=-2.12 | -3.49 to -0.75 | 0.003 |
| LARS | Reverse-dipper vs dipper | *β*=-3.37 | -5.78 to -0.96 | 0.006 |
| LARS | Extreme-dipper vs dipper | *β*=-0.96 | -3.18 to 1.26 | 0.397 |
| Low LARS | Non-dipper vs dipper | OR=2.49 | 1.13-5.49 | 0.024 |
| Low LARS | Reverse-dipper vs dipper | OR=4.18 | 1.30-13.45 | 0.016 |
| Low LARS | Extreme-dipper vs dipper | OR=1.56 | 0.41-5.98 | 0.515 |

Note: Nighttime mean SBP replaced 24-hour mean SBP, and all other covariates were identical to those in Model 2. *β* denotes the linear regression coefficient, OR denotes the odds ratio from logistic regression, CI denotes confidence interval, and P denotes a two-sided *P* value. Because dipping rate is calculated from daytime and nighttime SBP, this analysis was used to evaluate attenuation after adjustment for absolute nighttime pressure load; independent causal contributions require further evaluation in longitudinal studies and dedicated designs. The maximum VIF was 3.42. vs, comparison of the former group with the latter group; 95% CI, 95% confidence interval; LARS, left atrial reservoir strain; SBP, systolic blood pressure; VIF, variance inflation factor.

**Supplementary Table 8. Firth penalized-likelihood logistic sensitivity analysis**

| **Model** | **Contrast** | **Firth OR** | **95% CI** | ***P* value** |
| --- | --- | --- | --- | --- |
| Model 2 | Non-dipper vs dipper | 3.55 | 1.78-7.07 | <0.001 |
| Model 2 | Reverse-dipper vs dipper | 7.72 | 2.88-20.69 | <0.001 |
| Model 2 | Extreme-dipper vs dipper | 1.99 | 0.61-6.47 | 0.253 |
| Model 3 | Non-dipper vs dipper | 3.10 | 1.54-6.24 | 0.002 |
| Model 3 | Reverse-dipper vs dipper | 6.21 | 2.31-16.69 | <0.001 |
| Model 3 | Extreme-dipper vs dipper | 1.88 | 0.57-6.18 | 0.299 |

Note: Firth penalized-likelihood logistic regression was used. The covariates in Models 2 and 3 were identical to those in Table 5. CI, confidence interval; LARS, left atrial reservoir strain; OR, odds ratio; *P*, two-sided *P* value; vs, comparison of the former group with the latter group; 95% CI, 95% confidence interval; Firth penalized-likelihood method, a method used to reduce estimation bias in small-sample or rare-event logistic regression.

**Supplementary Table 9. Reproducibility of left atrial strain parameters**

| **Variable** | **Intraobserver ICC** | **95% CI** | ***P* value** | **Interobserver ICC** | **95% CI** | ***P* value** |
| --- | --- | --- | --- | --- | --- | --- |
| LARS | 0.97 | 0.95-0.98 | <0.001 | 0.93 | 0.88-0.96 | <0.001 |
| Left atrial conduit strain | 0.94 | 0.89-0.97 | <0.001 | 0.90 | 0.83-0.94 | <0.001 |
| Left atrial contraction strain | 0.91 | 0.84-0.95 | <0.001 | 0.87 | 0.79-0.92 | <0.001 |

Note: A random sample of 50 patients was used. Intraobserver agreement was assessed using a two-way mixed-effects, absolute-agreement, single-measure ICC [ICC(A,1)]; interobserver agreement was assessed using a two-way random-effects, absolute-agreement, single-measure ICC [ICC(2,1)]. CI, confidence interval; ICC, intraclass correlation coefficient; LARS, left atrial reservoir strain; *P*, two-sided *P* value; 95% CI, 95% confidence interval.
